# Velocity-Sensor-Based Dual-ESO Reconstruction for Feedforward–Feedback Low-Frequency Active Micro-Vibration Isolation

**DOI:** 10.3390/s26154715

**Published:** 2026-07-24

**Authors:** Zhenyu Fan, Yong Xie, Yuzhang Zhu, Yi Le, Yifei Zhang, Rui Xiu, Xindong Liang, Liang Zhang, Jianjun Jia

**Affiliations:** 1 Key Laboratory of Space Active Opto-Electronics Technology, Shanghai Institute of Technical Physics, Chinese Academy of Sciences, Shanghai 200083, China; fanzhenyu221@mails.ucas.ac.cn (Z.F.); ghostxy1987@hotmail.com (Y.X.); zhuyuzhang23@mails.ucas.ac.cn (Y.Z.); zhangyifei@mail.sitp.ac.cn (Y.Z.); xr090511@163.com (R.X.); jjjun10@mail.sitp.ac.cn (J.J.); 2 University of Chinese Academy of Sciences, Beijing 100049, China; leyi24@mails.ucas.ac.cn (Y.L.); liangxindong@ucas.ac.cn (X.L.); 3 Hefei National Laboratory, Hefei 230088, China; 4 Hangzhou Institute for Advanced Study, Hangzhou 310024, China

**Keywords:** low-frequency active micro-vibration isolation, dual extended state observer, state reconstruction, equivalent-displacement feedback, base-motion feedforward

## Abstract

Low-frequency base vibration transmitted through supporting platforms can degrade the stability of precision payloads, optical instruments, and inertial measurement systems. In velocity-sensor-based active isolation platforms, displacement-related feedback states are unavailable. Direct velocity integration can drift under sensor bias, while raw lower-platform velocity feedforward can introduce measurement noise and out-of-band components. This paper proposes a dual extended state observer (ESO) reconstruction method combining equivalent-displacement feedback and lower-platform feedforward. The upper-platform ESO reconstructs feedback velocity and a bounded equivalent-displacement state, while the lower-platform ESO provides a smoothed velocity reference for feedforward compensation. The method was implemented on a plate-type active vibration isolation platform and evaluated using lower-to-upper-platform acceleration transmissibility over 0.1–10 Hz. Across three repeated 200 s records, the proposed ESO feedback–feedforward condition achieved an integrated input–output suppression ratio of 38.67±0.46 dB, reduced the upper-platform output acceleration RMS to $\left(1.31\pm 0.09\right)\times 10^{-7}$ g, and provided a 48.92±2.12 dB RMS reduction relative to the passive baseline. Direct nominal–measured comparisons further showed that the reduced model captured the dominant passive and feedback-controlled dynamics. These results demonstrate that low-frequency active micro-vibration isolation can be achieved using only velocity measurements, without additional displacement sensors.

## 1. Introduction

Low-frequency micro-vibration is a persistent disturbance for spacecraft precision payloads, high-resolution optical instruments, atomic-interferometry systems, ultra-precision manufacturing equipment, and other high-stability platforms. Disturbances generated by reaction wheels, control-moment gyroscopes, cryocoolers, and flexible appendages can degrade pointing stability, imaging quality, frequency stability, and positioning accuracy [1,2,3,4]. For spaceborne infrared instruments, external micro-vibration can affect the optical-path-difference velocity uniformity of translational Fourier-transform interferometers [5]. On-orbit degradation and environmental variation can also induce nonlinear responses that reduce the calibration accuracy of hyperspectral infrared sounders [6]. Passive isolation is effective above its corner frequency, but its resonance amplification and limited low-frequency attenuation restrict its use when the target band extends to the sub-10 Hz range. Active isolation is therefore widely adopted in low-frequency micro-vibration control because it can reshape the apparent stiffness and damping of the isolation stage and suppress vibration transmission from the lower platform to the upper platform in the target band [1,2,7,8,9,10,11]. Broader vibration-control studies have also explored sky-hook positive-real-network ISD suspensions for vehicle seats and 1:2 internal-resonance mechanisms that synchronize broadband energy harvesting with vibration mitigation [12,13].

The feedback variable is a central issue in low-frequency active micro-vibration isolation. Velocity feedback, often interpreted as sky-hook or damping-like control, is useful for reducing resonance amplification and improving dynamic damping [14]. However, residual low-frequency motion is also affected by the stiffness-like component of the active control law. Displacement or position feedback can provide this low-frequency regulation by modifying the apparent dynamic stiffness, and related ideas have been used in blended stiffness control, absolute–relative feedback, inertial-actuator displacement feedback, piezoelectric active isolation, and sensor-fusion-based positioning and vibration suppression [9,15,16,17,18]. In platforms equipped only with velocity sensors, this displacement-type state is unavailable. Direct integration of the measured velocity is not a satisfactory substitute, because sensor bias and very-low-frequency noise can accumulate into drift, and high-frequency noise may be introduced into the actuation command through the feedback loop [18,19,20]. A practical velocity-sensor-based controller therefore requires a displacement-like feedback state that can provide low-frequency regulation without relying on an additional displacement sensor or direct velocity integration.

Feedforward compensation addresses a complementary part of the isolation problem. When lower-platform motion can be measured, feedforward control can reduce measurable lower-platform vibration transmission before it appears in the upper-platform response. Disturbance feedforward, adaptive feedforward, self-tuning feedforward, and composite feedforward–feedback schemes have been proved to improve active vibration isolation when a lower-platform reference is available [11,21,22,23]. The effectiveness of such compensation, however, depends strongly on the quality of the feedforward reference. Raw lower-platform velocity can contain sensor noise, out-of-band components, transfer-path mismatch, and phase errors; injecting this signal may directly reduce compensation accuracy. Thus, the lower-platform motion reference should be reconstructed as a control-oriented feedforward state rather than used as an unprocessed sensor signal.

Observer-based methods provide a systematic route to improve state availability and signal quality under limited sensing. Qian et al. combined a disturbance observer with linear quadratic regulation to reject floor-induced disturbances in optical reference cavities [4]. Beijen et al. developed disturbance feedforward and self-tuning multiple-input multiple-output (MIMO) feedforward strategies for hard-mounted active isolators, emphasizing measurable base disturbances and isolator internal dynamics [22,23]. Wang et al. used sensor fusion to support simultaneous positioning and vibration suppression in a multi-degree-of-freedom isolator [18]. Shi et al. introduced an improved linear ESO for a maglev inertially stabilized platform to enhance isolation under uncertainty [24]. In parallel, active disturbance rejection control (ADRC) and ESO studies have provided systematic approaches for bandwidth tuning, implementation, noise–performance trade-off analysis, and stability assessment of observer-based control systems [25,26,27,28,29,30,31].

These studies demonstrate the value of observers for disturbance rejection, state estimation, and feedforward compensation. For a velocity-sensor-only active micro-vibration isolation platform, however, the velocity measurements from the upper and lower platforms serve different control purposes. The upper-platform measurement must generate feedback states for damping and low-frequency equivalent-displacement regulation, whereas the lower-platform measurement must provide a smooth velocity reference for feedforward compensation. A single generic observer or conventional low-pass preprocessing cannot simultaneously satisfy these two control requirements.

To address this task separation, this paper develops a velocity-sensor-based dual-ESO state-reconstruction method for low-frequency active micro-vibration isolation. Different reconstruction tasks are assigned to the two velocity measurements: the upper-platform ESO generates the feedback velocity and a bounded equivalent-displacement state for low-frequency regulation, whereas the lower-platform ESO provides a smoothed velocity reference for feedforward compensation. The observer bandwidths are selected by jointly considering transmissibility reduction, noise-to-command gain, and nominal feedback-loop stability, and the reconstructed states are incorporated into a composite feedback–feedforward control law. The method is validated experimentally on a plate-type active isolation platform under five conditions: passive isolation, low-pass-filtered PI feedback, low-pass-filtered PI feedback with proportional feedforward, ESO feedback, and ESO feedback with ESO feedforward.

## 2. System Modeling and Problem Formulation

### 2.1. Plate-Type Active Isolation Platform

**Figure 1:**
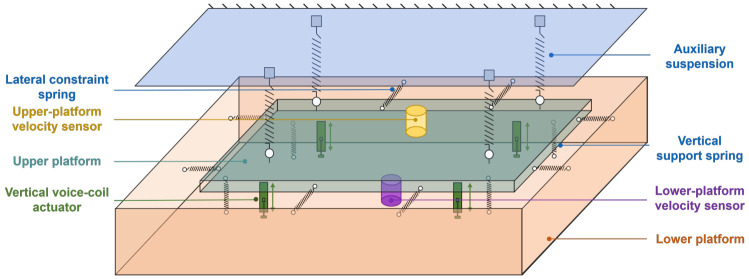
Schematic of the vertical control configuration of the plate-type active isolation platform.

The experimental platform consists of an upper platform, a lower platform placed on a marble base, vertical support springs, auxiliary suspension elements, velocity sensors mounted on the upper and lower platforms, distributed voice-coil actuators, and lateral constraint springs. The upper platform serves as the isolated plate carrying the payload, whereas the lower platform serves as the base plate and follows the motion of the marble base. The upper-platform velocity sensor is used for feedback-state reconstruction, while the lower-platform velocity sensor provides the base-motion reference for feedforward compensation. Four vertical voice-coil actuators are arranged near the plate corners to generate the active control force. This work focuses on low-frequency active micro-vibration isolation in the vertical (z-axis) direction; therefore, the modeling, control design, and experimental evaluation are all conducted along this direction, as shown in Figure 1.

The upper platform is supported relative to the lower platform by the vertical support springs, and it is also connected to the ground-side suspension reference through auxiliary suspension elements. Lateral springs provide horizontal constraint and are not included in the vertical model.

The reduced model is obtained by small-displacement vertical linearization around the static equilibrium. The lateral constraint springs mainly restrict horizontal motion, so their first-order vertical stiffness and damping contributions are negligible in the nominal vertical model. Any residual geometric coupling is represented by the unmodeled term *d*.

As shown in Figure 1, the vertical support branch between the upper and lower platforms is represented by the equivalent stiffness and damping $K_{s}$ and $C_{s}$, while the auxiliary suspension branch is represented by $K_{h}$ and $C_{h}$ before low-frequency simplification. The resulting vertical dynamics are given by Equation (1):

(1) $$m{\ddot{z}}_{p}+C_{s}\left({\dot{z}}_{p}-{\dot{z}}_{b}\right)+K_{s}\left(z_{p}-z_{b}\right)+C_{h}\left({\dot{z}}_{p}-{\dot{z}}_{h}\right)+K_{h}\left(z_{p}-z_{h}\right)=u+d,$$

where $z_{p}$ is the absolute vertical displacement of the upper platform, $z_{b}$ is the displacement coordinate of the lower platform, $z_{h}$ is the suspension-reference displacement, *m* is the equivalent mass of the upper platform and payload, and *u* is the vertical active control force.

A low-frequency equivalent model is adopted for the 0.1–10 Hz vertical rigid-body band considered in this study. The suspension frame is rigidly connected to the ground-side support, and its first structural resonance frequency is approximately 166 Hz, which is far above the target band. Therefore, within the target band, the suspension-reference displacement is approximated as equal to the lower-platform displacement, as stated in Equation (2):

(2) $$z_{h}\approx z_{b},$$

Using Equations (1) and (2), the two vertical passive branches are combined into equivalent stiffness and damping terms, and the reduced vertical dynamic model is given by Equation (3):

(3) $$m{\ddot{z}}_{p}+c\left({\dot{z}}_{p}-{\dot{z}}_{b}\right)+k\left(z_{p}-z_{b}\right)=u+d,$$

The corresponding equivalent coefficients are defined by Equation (4):

(4) $$c=C_{s}+C_{h},\;k=K_{s}+K_{h}.$$

This low-frequency equivalent model is used for controller design and transmissibility analysis in the 0.1–10 Hz target band.

**Figure 2:**
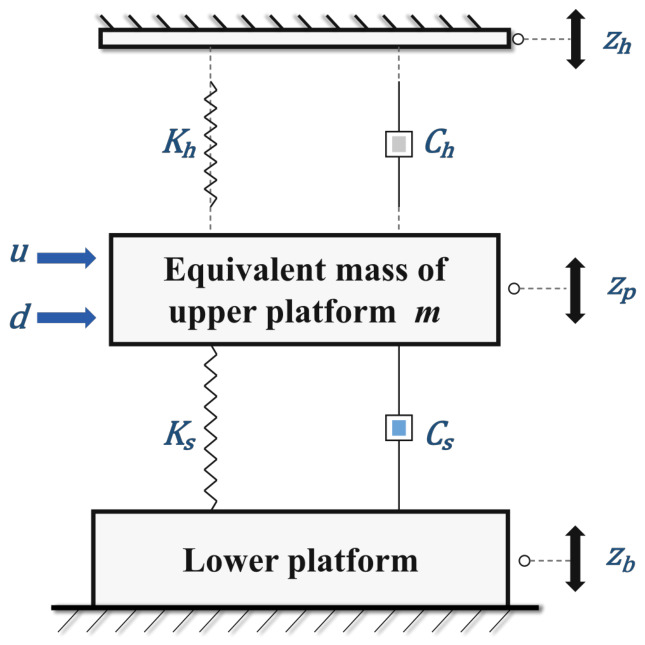
Vertical single-degree-of-freedom equivalent dynamic model.

The resulting single-degree-of-freedom representation is shown in Figure 2; the upper mass is connected to the lower platform through $K_{s}$ and $C_{s}$ and to the suspension reference through $K_{h}$ and $C_{h}$.

### 2.2. Frequency-Domain Relations Under Velocity Measurement

Let $V_{p}\left(s\right)$, $V_{b}\left(s\right)$, and U(s) denote the Laplace transforms of the upper-platform velocity, lower-platform velocity, and active control force, respectively. By neglecting *d* in the nominal input–output model, the lower-platform-to-upper-platform velocity transfer function is

(5) $$G_{b}\left(s\right)=\frac{V_{p}\left(s\right)}{V_{b}\left(s\right)}=\frac{cs+k}{ms^{2}+cs+k},$$

and the control-force-to-platform-velocity transfer function is

(6) $$G_{u}\left(s\right)=\frac{V_{p}\left(s\right)}{U(s)}=\frac{s}{ms^{2}+cs+k}.$$

Equations (5) and (6) represent the lower-to-upper platform transmission path and the actuator force-to-motion path, respectively. Absolute-velocity feedback can increase the apparent damping and reduce the resonance peak. However, without an available displacement state, velocity feedback alone cannot provide sufficient low-frequency stiffness regulation [18,19]. Lower-platform velocity feedforward is additionally affected by transfer-path error, phase lag, and measurement noise [22,23].

The measured velocity signals are modeled by Equation (7):

(7) $$y_{p}=v_{p}+n_{p},\;y_{b}=v_{b}+n_{b},$$

where $y_{p}$ and $y_{b}$ denote the velocity sensor outputs on the upper and lower platforms, respectively; $v_{p}$ and $v_{b}$ are the corresponding true velocities; and $n_{p}$ and $n_{b}$ represent the measurement noises. The upper-platform velocity measurement contains the main feedback information. However, it does not directly provide the displacement-related state required for low-frequency regulation. A straightforward integration of $y_{p}$ may suffer from accumulated errors caused by low-frequency bias and drift. The lower-platform velocity measurement provides a measurable reference of the base motion, but direct feedforward of the raw signal may introduce measurement noise and out-of-band components into the control command. These limitations motivate the use of an upper-platform ESO for feedback-state reconstruction and a lower-platform ESO for smoothed lower-platform velocity reconstruction.

### 2.3. Control Problem

The control objective is to suppress the upper-platform vibration in the 0.1–10 Hz band using only the upper- and lower-platform velocity measurements. The controller should provide damping and low-frequency regulation while avoiding direct integration of the raw feedback velocity, excessive noise amplification, and direct raw-signal feedforward. The active control force is decomposed into feedback and feedforward components, as defined by Equation (8):

(8) $$u=u_{\mathrm{fb}}+u_{\mathrm{ff}},$$

where $u_{\mathrm{fb}}$ is the feedback component and $u_{\mathrm{ff}}$ is the feedforward component. The feedback path uses the reconstructed upper-platform velocity and equivalent-displacement feedback state to reduce the resonance peak and improve low-frequency regulation. The feedforward path uses a smoothed lower-platform velocity state to attenuate measurable lower-platform vibration transmission.

## 3. Dual-ESO State Reconstruction and Composite Control

### 3.1. Overall Framework

The proposed method uses the two measured velocity signals for two control-oriented reconstruction tasks. The upper-platform velocity measurement is used to reconstruct the feedback states, namely, the upper-platform velocity ${\hat{v}}_{p}$ and a bounded equivalent-displacement feedback state $z_{e}$. The lower-platform velocity measurement is used to reconstruct a smoothed lower-platform velocity state ${\hat{v}}_{b}$ for lower-platform feedforward. Thus, the two ESOs are assigned different control roles: the upper ESO improves the quality of the reconstructed feedback states for damping enhancement and low-frequency regulation, whereas the lower ESO improves the quality of the feedforward reference for compensating measurable lower-platform vibration.

**Figure 3:**
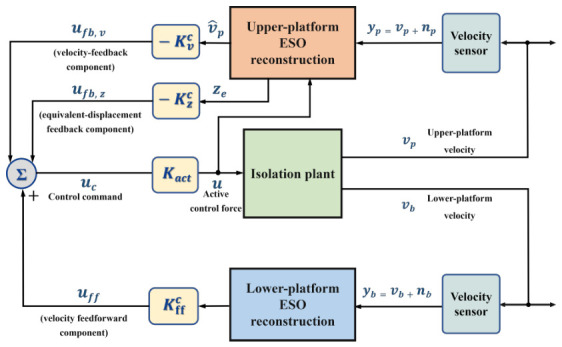
Overall dual-ESO control framework for low-frequency active micro-vibration isolation. The variable $z_{e}$ denotes a controller-internal equivalent-displacement feedback state, rather than a directly measured displacement.

Figure 3 shows the resulting composite controller. The feedback command consists of a velocity-feedback component generated from ${\hat{v}}_{p}$ and an equivalent-displacement feedback component generated from the feedback state $z_{e}$. The feedforward command is generated from the lower-platform ESO output ${\hat{v}}_{b}$. These command-side components are summed to form the controller command $u_{c}$, which is then converted into the physical actuator force *u* through the calibrated actuator chain. This distinction is retained in the following analysis because the controller gains are implemented on the command side, whereas the plant model uses physical force as its input.

### 3.2. Upper-Platform ESO for Feedback-State Reconstruction

For feedback-state reconstruction, the upper-platform velocity dynamics in (3) are written in the control-oriented form given by Equation (9):

(9) $${\dot{v}}_{p}=b_{0}u+d_{p},$$

where $b_{0}$ is the nominal force-to-acceleration coefficient, and $d_{p}$ represents the lumped contribution of passive dynamics, residual excitation, and unmodeled terms in the upper-platform velocity dynamics. Since *u* is defined as the active control force in the equivalent model, the observer uses $b_{0}=1/m_{0}$, where $m_{0}$ is the nominal upper-platform mass. Although the ESO also estimates the lumped disturbance $d_{p}$, the estimated disturbance ${\hat{d}}_{p}$ is not directly introduced into the control law for disturbance compensation, because the present design emphasizes robust feedback-state reconstruction rather than aggressive disturbance cancellation. Instead, the upper-platform ESO is mainly used to reconstruct the feedback states required by the velocity-feedback and equivalent-displacement feedback paths.

The velocity-side upper ESO is defined by Equation (10):

(10) $$\begin{array}{cc} {\dot{\hat{v}}}_{p} & ={\hat{d}}_{p}+b_{0}u+\beta_{p1}\left(y_{p}-{\hat{v}}_{p}\right),\\ {\dot{\hat{d}}}_{p} & =\beta_{p2}\left(y_{p}-{\hat{v}}_{p}\right), \end{array}$$

where ${\hat{v}}_{p}$ is the reconstructed upper-platform velocity, and $\beta_{p1}$ and $\beta_{p2}$ are observer gains. The upper-ESO gains are parameterized by the observer bandwidth $\omega_{op}$ according to Equation (11):

(11) $$\beta_{p1}=2\omega_{op},\;\beta_{p2}=\omega_{op}^{2}.$$

The measured velocity $y_{p}$ enters the observer through the innovation term $y_{p}-{\hat{v}}_{p}$. The resulting ${\hat{v}}_{p}$ is used in the damping-feedback path, whereas ${\hat{d}}_{p}$ is retained as an internal observer state and is not directly introduced into the control law for disturbance compensation.

Direct integration of $y_{p}$ is sensitive to sensor bias and quasi-static drift. To form a low-frequency displacement-like feedback variable without directly integrating the raw velocity signal, the reconstructed velocity is passed through an ESO-assisted reconstruction channel,

(12) $${\dot{z}}_{e}=-\omega_{z}z_{e}+{\hat{v}}_{p}+\alpha_{p}\left(y_{p}-{\hat{v}}_{p}\right),$$

where $z_{e}$ is the controller-internal equivalent-displacement feedback state, $\alpha_{p}$ is an error-injection coefficient, and $\omega_{z}$ is a very-low-frequency drift-suppression rate. The state $z_{e}$ is therefore not a measured absolute displacement and is not intended to represent the long-term platform position. It is a bounded feedback state designed to provide low-frequency regulation while avoiding the unbounded drift that would result from raw velocity integration.

Solving (12) gives the exponentially weighted reconstruction

(13) $$z_{e}\left(t\right)=e^{-\omega_{z}t}z_{e}\left(0\right)+\int_{0}^{t}e^{-\omega_{z}\left(t-\tau \right)}\left[{\hat{v}}_{p}\left(\tau \right)+\alpha_{p}\left(y_{p}\left(\tau \right)-{\hat{v}}_{p}\left(\tau \right)\right)\right]d\tau .$$

Equation (13) shows that $z_{e}$ is obtained by leaky integration of the reconstructed velocity and the measurement residual. The exponential kernel $e^{-\omega_{z}\left(t-\tau \right)}$ gives the reconstruction channel finite memory, preventing low-frequency bias and drift from accumulating without bound as in a pure integrator. In this reconstruction, ${\hat{v}}_{p}$ provides the main velocity information, while $\alpha_{p}\left(y_{p}-{\hat{v}}_{p}\right)$ introduces a weighted residual correction. Thus, $z_{e}$ acts as a bounded displacement-related feedback state for low-frequency regulation, without directly integrating the raw velocity measurement.

The same reconstruction mechanism can be expressed in the frequency domain. Neglecting the initial condition and setting the feedback-force input to zero for signal-path analysis, the velocity measurement-to-equivalent-displacement-state transfer function is

(14) $$H_{vp}\left(s,\omega_{op}\right)=\frac{{\hat{V}}_{p}\left(s\right)}{Y_{p}\left(s\right)}=\frac{2\omega_{op}s+\omega_{op}^{2}}{s^{2}+2\omega_{op}s+\omega_{op}^{2}}.$$

For compactness, $H_{vp}\left(s\right)$ denotes $H_{vp}\left(s,\omega_{op}\right)$ in the following equations. Combining (14) with (12), the velocity measurement-to-equivalent-displacement channel is

(15) $$H_{z}\left(s\right)=\frac{Z_{e}\left(s\right)}{Y_{p}\left(s\right)}=\frac{H_{vp}\left(s\right)+\alpha_{p}\left[1-H_{vp}\left(s\right)\right]}{s+\omega_{z}}.$$

Compared with a pure integrator, (15) has a finite gain as s→0 due to the leakage term $\omega_{z}$. Therefore, the reconstructed state avoids unbounded low-frequency accumulation, while the ESO velocity estimate and the measurement residual determine the reconstruction behavior in the target band.

### 3.3. Lower-Platform ESO for Feedforward-State Reconstruction

The lower-platform velocity is a measurable motion reference for feedforward compensation. However, using the raw measured lower-platform velocity directly can transmit sensor noise and out-of-band components into the actuator command. The lower-platform ESO is therefore used to reconstruct a smoothed lower-platform velocity state for feedforward control, rather than to track every high-frequency fluctuation of the raw measurement.

The lower-platform ESO is defined by Equation (16):

(16) $$\begin{array}{cc} {\dot{\hat{v}}}_{b} & ={\hat{a}}_{b}+\beta_{b1}\left(y_{b}-{\hat{v}}_{b}\right),\\ {\dot{\hat{a}}}_{b} & =\beta_{b2}\left(y_{b}-{\hat{v}}_{b}\right), \end{array}$$

where ${\hat{v}}_{b}$ is the reconstructed lower-platform velocity, ${\hat{a}}_{b}$ is the auxiliary acceleration-related state for the lower-platform channel, and $y_{b}$ is the lower-platform velocity measurement. The corresponding transfer function from $y_{b}$ to ${\hat{v}}_{b}$ is given by Equation (17):

(17) $$H_{b}\left(s,\omega_{ob}\right)=\frac{{\hat{V}}_{b}\left(s\right)}{Y_{b}\left(s\right)}=\frac{2\omega_{ob}s+\omega_{ob}^{2}}{s^{2}+2\omega_{ob}s+\omega_{ob}^{2}},$$

The observer gains are parameterized by Equation (18):

(18) $$\beta_{b1}=2\omega_{ob},\;\beta_{b2}=\omega_{ob}^{2}.$$

The bandwidth $\omega_{ob}$ determines the trade-off between feedforward responsiveness and noise attenuation. A larger $\omega_{ob}$ makes the feedforward signal closer to the raw lower-platform velocity, whereas a smaller $\omega_{ob}$ gives a smoother command reference but may reduce compensation bandwidth.

### 3.4. Composite Control Law

The command-side composite control law is given by Equation (19):

(19) $$u_{c}=-K_{z}^{c}z_{e}-K_{v}^{c}{\hat{v}}_{p}+K_{\mathrm{ff}}^{c}{\hat{v}}_{b},$$

where $K_{v}^{c}$, $K_{z}^{c}$, and $K_{\mathrm{ff}}^{c}$ are the command-side velocity-feedback, equivalent-displacement-feedback, and feedforward gains, respectively. The physical force applied to the platform is given by Equation (20):

(20) $$u=K_{\mathrm{act}}u_{c},$$

where $K_{\mathrm{act}}$ is the calibrated total actuator-chain coefficient. The force-equivalent gains used in the frequency-domain plant analysis are therefore defined by Equation (21):

(21) $${\bar{K}}_{v}=K_{\mathrm{act}}K_{v}^{c},\;{\bar{K}}_{z}=K_{\mathrm{act}}K_{z}^{c},\;{\bar{K}}_{\mathrm{ff}}=K_{\mathrm{act}}K_{\mathrm{ff}}^{c}.$$

This separation keeps the implementation parameters and the force-domain plant model consistent: the controller is tuned and implemented as a command-voltage law, while the transmissibility and stability analyses use the equivalent physical force acting on the plant.

### 3.5. Bandwidth Selection and Frequency-Domain Trade-Off

The bandwidth study uses the low-frequency equivalent model in (3). The model parameters are m=48 kg, $f_{n}=2.30$ Hz, $k=1.00\times 10^{4}$ N/m, and c=36 N·s/m, where $f_{n}$ denotes the identified vertical resonance frequency. The actuator coefficient is $K_{\mathrm{act}}=1.16$ N/V. Unless otherwise stated, the command-side gains are kept fixed at $K_{v}^{c}=18$, $K_{z}^{c}=100$, and $K_{\mathrm{ff}}^{c}=120$, so that the plotted trends mainly reflect the observer-bandwidth effects rather than controller retuning.

The upper ESO affects the reconstructed feedback states and the transmission of measurement noise to the feedback command. Its candidate bandwidths are listed in Equation (22):

(22) $$\omega_{op}\in \left\{50,100,200,300,500\right\}\;\mathrm{rad}/s.$$

This range covers observer settings from relatively low to high bandwidth in relation to the 0.1–10 Hz target isolation band, while also extending the observer dynamics beyond the main resonance region. The sweep is used to evaluate how the upper-ESO bandwidth influences feedback-state reconstruction, resonance suppression, and noise amplification in the feedback command.

The lower ESO is used to generate the feedforward reference from the lower-platform velocity measurement. Since this reference is injected into the actuator command through the feedforward path, an excessively large lower-ESO bandwidth may transmit measurement noise and out-of-band lower-platform motion to the control command. Therefore, the smaller bandwidth range in Equation (23) is examined:

(23) $$\omega_{ob}\in \left\{0.02,0.04,0.05,0.06,0.08\right\}\;\mathrm{rad}/s.$$

This range corresponds approximately to 0.003–0.013 Hz and is selected below the 0.1–10 Hz target isolation band to obtain a smoothed lower-platform feedforward reference. The sweep evaluates the trade-off between noise suppression and feedforward responsiveness: a smaller $\omega_{ob}$ reduces noise transmission but may attenuate or delay useful lower-platform motion information, whereas a larger $\omega_{ob}$ improves responsiveness at the cost of increased feedforward-command noise. The equivalent-displacement feedback-state reconstruction uses $\alpha_{p}=0.05$ and $\omega_{z}=0.1257$ rad/s, with $\omega_{z}$ corresponding to 0.02 Hz to provide finite memory below the target isolation band.

For the frequency-domain study, the feedback and feedforward controllers are written in Equations (24) and (25), respectively:

(24) $$\begin{array}{cc} C_{\mathrm{fb}}\left(s\right) & ={\bar{K}}_{v}H_{vp}\left(s\right)+{\bar{K}}_{z}H_{z}\left(s\right), \end{array}$$

(25) $$\begin{array}{cc} C_{\mathrm{ff}}\left(s\right) & ={\bar{K}}_{\mathrm{ff}}H_{b}\left(s,\omega_{ob}\right). \end{array}$$

Here, $C_{\mathrm{fb}}\left(s\right)$ and $C_{\mathrm{ff}}\left(s\right)$ are expressed on the physical-force side. The corresponding command-side feedback controller, used only for noise-to-command evaluation, is given by Equation (26):

(26) $$C_{\mathrm{fb}}^{c}\left(s\right)=K_{v}^{c}H_{vp}\left(s\right)+K_{z}^{c}H_{z}\left(s\right).$$

The actuator force-to-velocity path used here is given by Equation (27):

(27) $$G_{u}\left(s\right)=\frac{s}{ms^{2}+cs+k},$$

The resulting upper-platform velocity relation is given by Equation (28):

(28) $$v_{p}\left(s\right)=G_{u}\left(s\right)\left\{P\left(s\right)v_{b}\left(s\right)-C_{\mathrm{ff}}\left(s\right)v_{b}\left(s\right)-C_{\mathrm{fb}}\left(s\right)v_{p}\left(s\right)\right\}.$$

Therefore, the passive, feedback-only, and feedback-plus-feedforward transmissibility models are obtained in Equations (29), (30), and (31), respectively:

(29) $$\begin{array}{cc} G_{\mathrm{psv}}\left(s\right) & =P\left(s\right)G_{u}\left(s\right), \end{array}$$

(30) $$\begin{array}{cc} G_{\mathrm{fb}}\left(s\right) & =\frac{P\left(s\right)G_{u}\left(s\right)}{1+G_{u}\left(s\right)C_{\mathrm{fb}}\left(s\right)}, \end{array}$$

(31) $$\begin{array}{cc} G_{\mathrm{fbff}}\left(s\right) & =\frac{\left[P\left(s\right)-C_{\mathrm{ff}}\left(s\right)\right]G_{u}\left(s\right)}{1+G_{u}\left(s\right)C_{\mathrm{fb}}\left(s\right)}. \end{array}$$

The magnitude of each transmissibility curve is reported in decibels using Equation (32):

(32) $$T\left(f\right)=20\log_{10}\left|G(j2\pi f)\right|.$$

To evaluate noise sensitivity, additive measurement noise is introduced at the corresponding velocity measurement input, while the external excitation is set to zero. For the feedback path, let $n_{p}$ denote the upper-platform velocity measurement noise and $v_{p,n}$ denote the noise-induced upper-platform velocity response. Following the same negative-feedback relation as in (28), the feedback-noise relation is given by Equation (33):

(33) $$v_{p,n}\left(s\right)=G_{u}\left(s\right)\left[-C_{\mathrm{fb}}\left(s\right)\left(v_{p,n}\left(s\right)+n_{p}\left(s\right)\right)\right].$$

Rearranging Equation (33) gives Equation (34):

(34) $$v_{p,n}\left(s\right)+n_{p}\left(s\right)=\frac{1}{1+G_{u}\left(s\right)C_{\mathrm{fb}}\left(s\right)}n_{p}\left(s\right).$$

The resulting command-side feedback signal is given by Equation (35):

(35) $$u_{\mathrm{fb},n}^{c}\left(s\right)=-C_{\mathrm{fb}}^{c}\left(s\right)\left(v_{p,n}\left(s\right)+n_{p}\left(s\right)\right),$$

Combining Equations (34) and (35) gives the upper-platform measurement noise-to-feedback-command transfer function in Equation (36):

(36) $$G_{n,\mathrm{fb}}\left(s\right)=\frac{u_{\mathrm{fb},n}^{c}\left(s\right)}{n_{p}\left(s\right)}=-\frac{C_{\mathrm{fb}}^{c}\left(s\right)}{1+G_{u}\left(s\right)C_{\mathrm{fb}}\left(s\right)}.$$

The negative sign follows the negative-feedback convention and does not affect the magnitude evaluation.

For the feedforward path, the lower-platform measurement noise is mapped to the command through the lower-ESO reconstruction and the command-side feedforward gain. The feedforward noise command is given by Equation (37):

(37) $$u_{\mathrm{ff},n}^{c}\left(s\right)=K_{\mathrm{ff}}^{c}H_{b}\left(s,\omega_{ob}\right)n_{b}\left(s\right),$$

Equation (38) then gives the lower-platform measurement noise-to-feedforward-command transfer function:

(38) $$G_{n,\mathrm{ff}}\left(s\right)=\frac{u_{\mathrm{ff},n}^{c}\left(s\right)}{n_{b}\left(s\right)}=K_{\mathrm{ff}}^{c}H_{b}\left(s,\omega_{ob}\right).$$

Equations (36) and (38) are not intended to provide a complete stochastic noise model. They are used only to compare how the selected observer bandwidths affect the command sensitivity to measurement noise.

**Figure 4:**
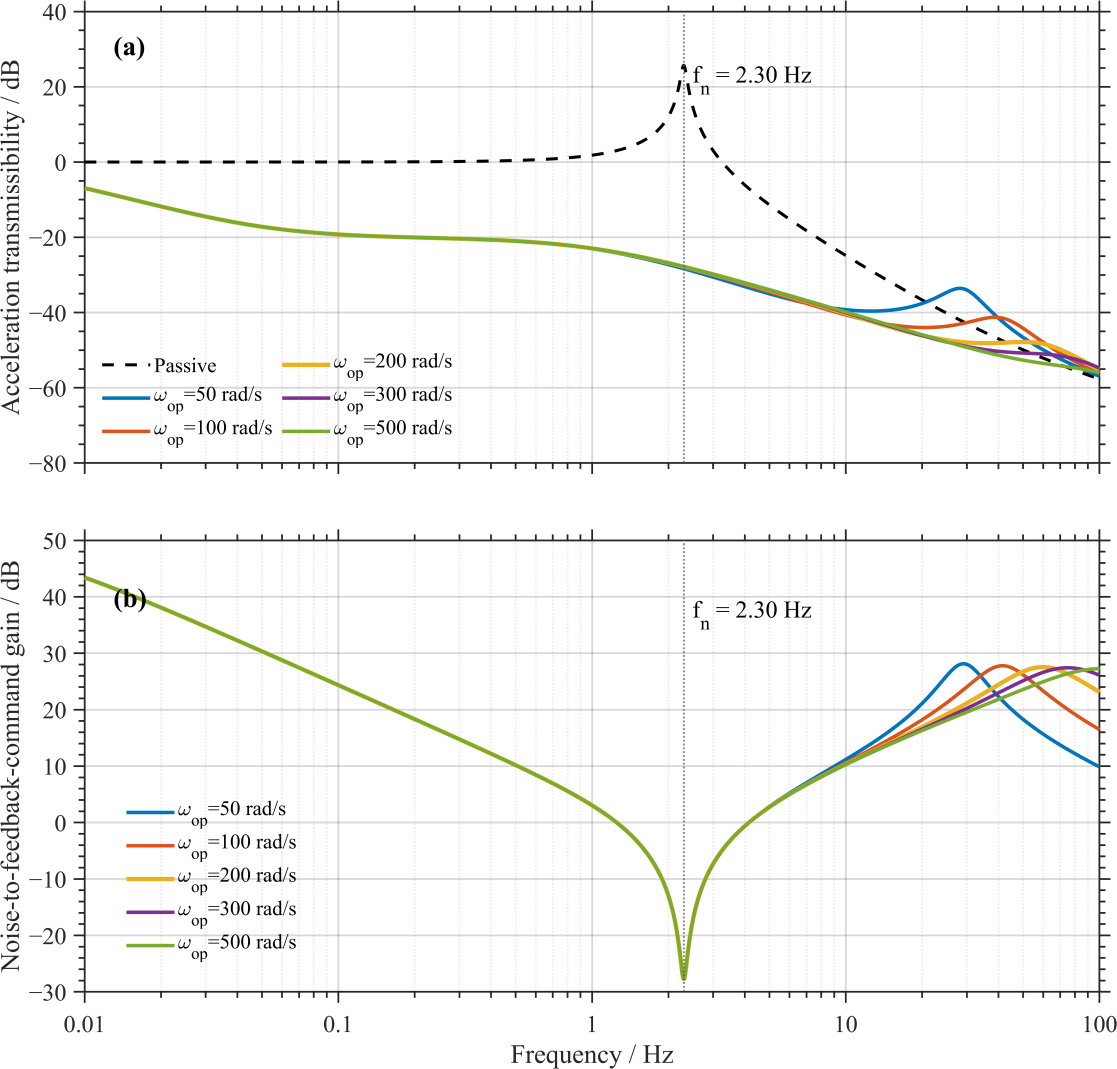
Upper-platform ESO bandwidth trade-off: (**a**) predicted acceleration transmissibility and (**b**) feedback-noise-to-command gain.

Figure 4a shows that increasing $\omega_{op}$ reduces the residual transmissibility rise above the mechanical resonance. However, Figure 4b also indicates that a faster upper ESO increases the noise-to-feedback-command gain at higher frequencies. This behavior reflects the trade-off introduced by placing the upper ESO inside the feedback loop: increasing the observer bandwidth improves feedback-state reconstruction and resonance suppression, but it also increases the transmission of sensor noise to the command channel. Among the plotted candidates, $\omega_{op}=50$ and 100 rad/s still exhibit a more pronounced high-frequency transmissibility rise, whereas $\omega_{op}=300$ and 500 rad/s provide limited additional benefit in the target band while increasing the high-frequency command sensitivity. Therefore, $\omega_{op}=200$ rad/s is selected as a balanced setting, providing strong resonance suppression while limiting the high-frequency sensor-noise transmission to the feedback command.

**Figure 5:**
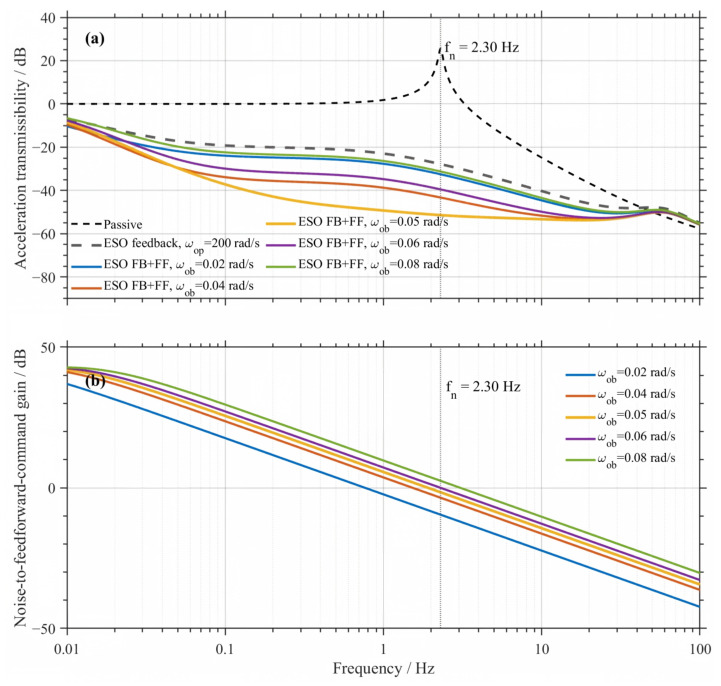
Lower-platform ESO bandwidth trade-off for lower-platform feedforward: (**a**) predicted acceleration transmissibility and (**b**) feedforward-noise-to-command gain.

Figure 5a shows that the feedforward path can provide additional low-frequency attenuation when the lower-ESO bandwidth is properly selected. Figure 5b shows the corresponding noise-to-feedforward-command gain. Unlike the upper ESO, the lower ESO is not placed in the feedback loop; instead, it reconstructs a smoothed lower-platform velocity reference for feedforward compensation. A smaller $\omega_{ob}$ provides stronger smoothing and lower noise transmission, but it may attenuate or delay useful lower-platform motion information. In contrast, a larger $\omega_{ob}$ allows the feedforward path to follow more of the measured lower-platform velocity, at the cost of increased sensitivity to measurement noise and out-of-band components. Therefore, $\omega_{ob}=0.05$ rad/s is selected as a balanced setting between feedforward compensation effectiveness and smoothing of the measured lower-platform velocity signal.

**Figure 6:**
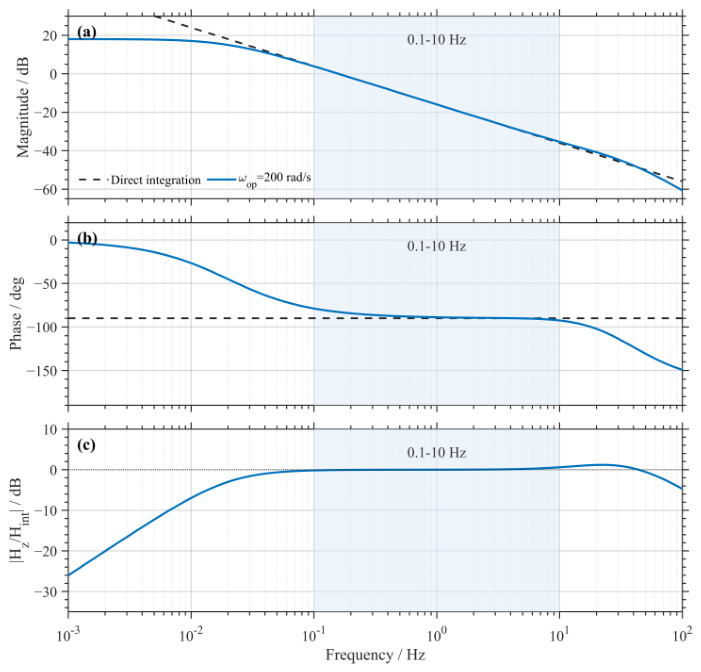
Selected velocity-to-equivalent-displacement reconstruction channel: (**a**) magnitude, (**b**) phase, and (**c**) gain relative to raw velocity integration. The blue shaded region denotes the 0.1–10 Hz target isolation band.

After selecting the observer bandwidths for the feedback and feedforward paths, the frequency-domain behavior of the equivalent-displacement reconstruction is further examined. Figure 6 shows that the selected reconstruction retains the integration-like behavior needed for displacement-related feedback in the 0.1–10 Hz band, while the leakage term limits the very-low-frequency gain and prevents unbounded bias accumulation.

### 3.6. Nominal Stability of the Selected Feedback Loop

The nominal stability of the selected feedback loop is evaluated using the open-loop transfer function

(39) $$L\left(s\right)=G_{u}\left(s\right)C_{\mathrm{fb}}\left(s\right),$$

where $C_{\mathrm{fb}}\left(s\right)$ includes the selected upper ESO reconstruction channels and the force-equivalent feedback gains. The feedforward channel is not included in (39) because it does not close an additional feedback loop around the upper-platform velocity.

**Figure 7:**
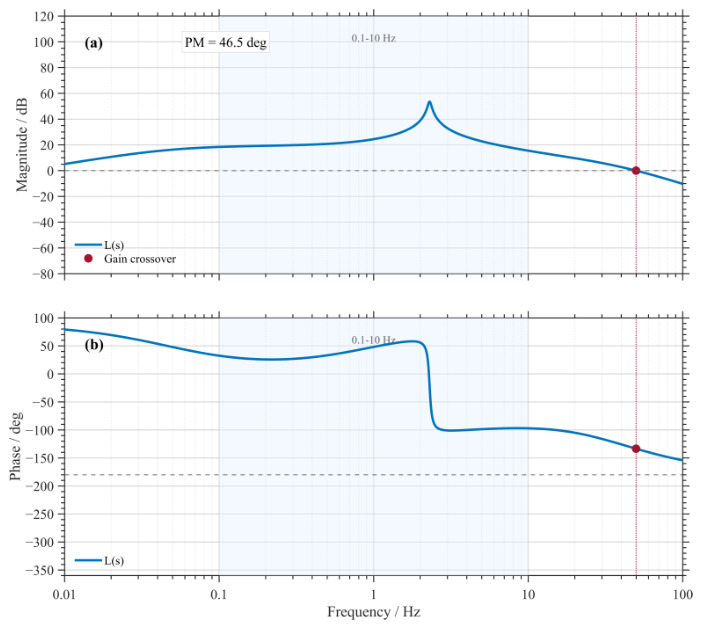
Nominal feedback-loop Bode plot for the selected ESO feedback controller. (**a**) Magnitude and (**b**) Phase. The phase margin is $46.5^{\circ }$. The blue shaded region denotes the 0.1–10 Hz target isolation band; the red marker and vertical dotted line indicate the gain crossover frequency.

As shown in Figure 7, the selected feedback loop has a phase margin of $46.5^{\circ }$. No finite phase-crossover frequency is found in the nominal model; therefore, the gain margin is not used as the primary plotted index. This result provides a nominal stability check for the fixed-gain feedback controller. Because the analysis is based on the single-degree-of-freedom equivalent model, it should be interpreted as a nominal loop-margin assessment rather than a complete robust-stability proof for all structural modes or payload variations.

### 3.7. Simulation Verification of Signal Reconstruction

The frequency-response and stability analysis justifies the selected parameters from the control-design perspective. A time-domain simulation is then used to verify whether the two ESOs produce the intended reconstructed signals under known bias and high-frequency noise components. This simulation is not used as the final evidence of isolation performance; rather, it is used to make the signal-reconstruction behavior observable before experimental validation.

For the lower-platform feedforward reference, the smoothing index was defined as the RMS reduction of the reconstruction residual:

(40) $$S_{b}=20\log_{10}\frac{\mathrm{RMS}\{y_{b}\left(t\right)-v_{b}^{\mathrm{sim}}\left(t\right)\}}{\mathrm{RMS}\{{\hat{v}}_{b}\left(t\right)-{\hat{v}}_{b}^{\mathrm{sim}}\left(t\right)\}},$$

where $v_{b}^{\mathrm{sim}}\left(t\right)$ is the noise-free lower-platform velocity used in the simulation and ${\hat{v}}_{b}^{\mathrm{sim}}\left(t\right)$ is the corresponding noise-free lower-ESO output. A positive $S_{b}$ means that the ESO output has a smaller residual RMS than the raw lower-platform measurement.

**Figure 8:**
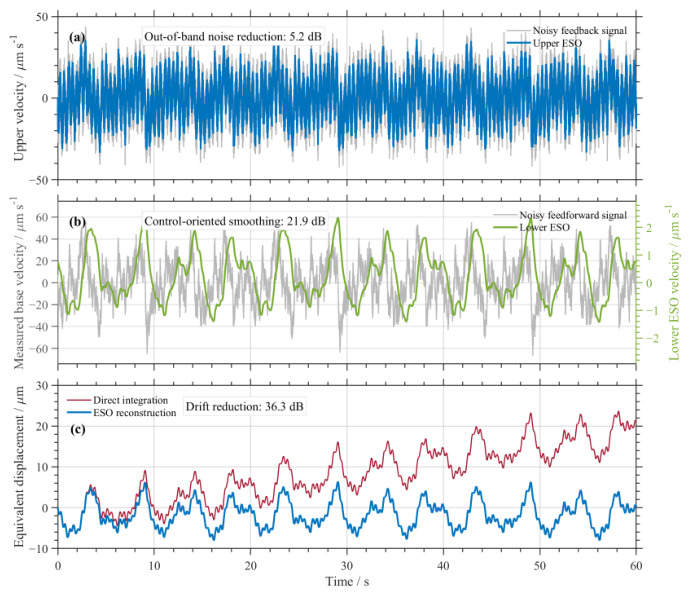
Simulation verification of dual-ESO signal reconstruction with the selected parameters: (**a**) noisy upper-platform feedback velocity and upper ESO velocity estimate; (**b**) noisy lower-platform velocity used for feedforward and lower-ESO smoothed lower-platform velocity state; (**c**) direct velocity integration and ESO equivalent-displacement reconstruction. A 60 s segment is shown.

Figure 8a shows that the upper ESO preserves the dominant feedback velocity while reducing out-of-band feedback-sensor noise by 5.2 dB in the 60–200 Hz band. Figure 8b shows that the lower ESO generates a smoothed lower-platform velocity state for feedforward control rather than tracking the raw noisy lower-platform signal point by point; using (40), the corresponding smoothing index is 21.9 dB. Figure 8c compares direct velocity integration with the ESO equivalent-displacement reconstruction. Consistent with Figure 6, direct integration accumulates drift, whereas the ESO-assisted reconstruction remains bounded and gives a drift reduction of 36.3 dB. These results support the intended roles of the two observers: feedback-state reconstruction, feedforward-reference smoothing, and suppression of direct-integration drift.

## 4. Experimental Validation and Results

### 4.1. Experimental Setup and Evaluation Metrics

**Figure 9:**
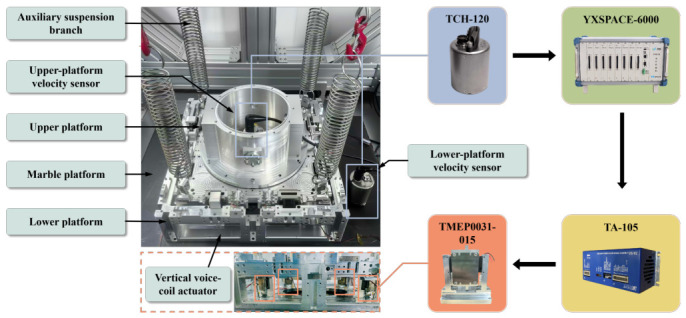
Active isolation platform and vertical test arrangement.

Experiments were performed on the same plate-type active vibration isolation platform used for low-frequency model identification and simulation parameterization. Figure 9 shows the experimental arrangement. The isolation platform was installed on a marble platform, which served as the laboratory foundation. Auxiliary suspension branches were used to provide gravity offloading for the upper platform during ground tests. Two TCH-120 broadband velocity sensors with a sensitivity of 754.3 V/(m/s) measured the absolute velocities of the upper and lower platforms. According to the manufacturer’s noise-floor curve for the 120 s model, the acceleration-noise density is on the order of $10^{-10}$ g/$\sqrt{\mathrm{Hz}}$ in the 0.1–10 Hz band, which is below the measured band-limited vibration levels used for transmissibility evaluation. The upper-platform velocity was used as the feedback measurement, whereas the lower-platform velocity was taken as the lower-platform input and feedforward reference. The control algorithm was implemented on a YXSPACE-6000 ground real-time simulation/control unit, which acquired the sensor signals and generated the actuator commands. The control command was converted into actuator force through four TMEP0031-015 voice-coil motors driven by TA-105 drivers. The actuator chain has a motor force constant of 5.8 N/A and a driver current gain of 0.05 A/V.

Five operating conditions were tested to separate the effects of feedback, feedforward, and ESO-based state reconstruction. The passive case was used as the uncontrolled baseline. The LPF-PI feedback case used conventional velocity feedback after 50 Hz low-pass preprocessing, and the LPF-PI plus P feedforward case further added proportional lower-platform velocity feedforward with the same preprocessing applied to both velocity channels. The ESO feedback case replaced the filtered feedback variables with the upper-ESO reconstructed velocity and equivalent-displacement feedback state, while the ESO feedback plus ESO feedforward case additionally used the lower ESO to reconstruct the lower-platform velocity feedforward state.

The 50 Hz low-pass filter was used only for the two LPF-PI cases. No additional 50 Hz low-pass filter was applied in the ESO feedback or ESO feedback–feedforward cases, where signal conditioning was performed by the ESO reconstruction itself. Thus, the five cases are compared as complete controller implementations with their respective signal-conditioning or reconstruction structures.

In all active cases, the velocity channels used for control passed through a common 0.001 Hz high-pass sensor-chain conditioning before command computation. This conditioning suppresses quasi-static sensor-chain offset far below the 0.1–10 Hz evaluation band; it is independent of the ESO equivalent-displacement reconstruction and is not used as postprocessing for isolation-performance evaluation.

All signals were sampled at 1000 Hz and evaluated in the 0.1–10 Hz band. Representative ASD and transmissibility curves were plotted from one 200 s record. Repeatability statistics were computed from three 200 s records. The acceleration amplitude spectral density (ASD) was obtained from the measured velocity records by standard spectral conversion and Welch-averaged periodograms with Hann windows and 50% overlap [32]. The input–output transmissibility was estimated from the lower-platform velocity input and upper-platform velocity output using the H1 frequency-response-function estimator [33,34]. For the measured data, the complex frequency response from lower-platform velocity to upper-platform velocity was denoted as ${\hat{G}}_{v_{p}v_{b}}\left(f\right)$. The tabulated transmissibility was computed as $T_{\mathrm{dB}}\left(f\right)=20\log_{10}\left|{\hat{G}}_{v_{p}v_{b}}\left(f\right)\right|$, consistent with (32) and the H1 estimator [33,34]. Band-mean transmissibility was obtained by averaging $T_{\mathrm{dB}}\left(f\right)$ over the specified frequency band.

Performance was quantified using three metrics: the band-limited upper-platform acceleration root-mean-square (RMS), the RMS reduction relative to the passive condition, and the integrated suppression ratio $\eta_{0.1-10}$. The integrated suppression ratio compares the lower-platform input energy with the upper-platform output energy over the 0.1–10 Hz band. It therefore evaluates isolation performance with respect to the measured ground-vibration input. Because the passive baseline amplifies the input near resonance, $\eta_{0.1-10}$ is used as the primary RMS-based measure of absolute input–output isolation, whereas the passive-relative RMS reduction is retained as a secondary measure of improvement over the uncontrolled resonant baseline. Each 200 s record contains 20 cycles at 0.1 Hz, the lower bound of the evaluation band, and therefore covers the full frequency range used for the RMS and integrated-suppression evaluations. The numerical results in Table 1 are reported as mean ± standard deviation over the three records.

### 4.2. Passive Baseline

**Figure 10:**
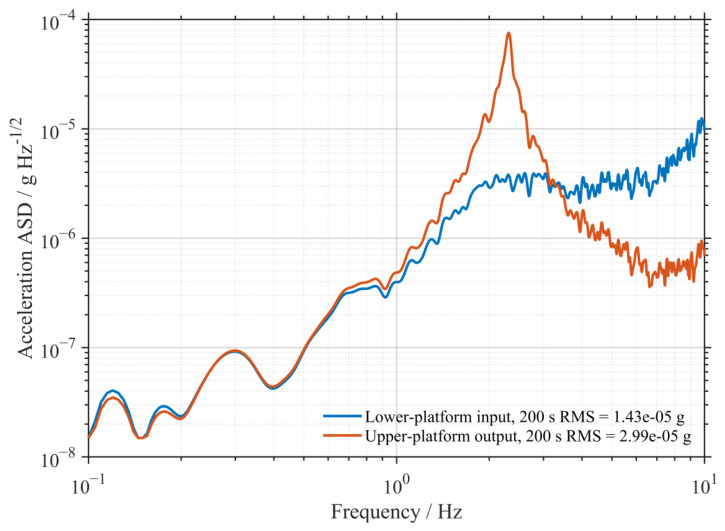
Passive baseline: lower-platform input and upper-platform output acceleration ASD in the 0.1–10 Hz band.

The passive input–output response was first measured under the same vertical ground-vibration environment. As shown in Figure 10, a representative 200 s passive record gave an integrated lower-platform input acceleration RMS of $1.4253\times 10^{-5}$ g and an integrated upper-platform output acceleration RMS of $2.9940\times 10^{-5}$ g in the 0.1–10 Hz band. The corresponding integrated suppression ratio, defined as $20\log_{10}\left({\mathrm{RMS}}_{\mathrm{in}}/{\mathrm{RMS}}_{\mathrm{out}}\right)$, was −6.45 dB. The negative value indicates that the upper-platform response was larger than the lower-platform input. This amplification was mainly caused by the strong response near the identified 2.30 Hz vertical resonance.

For the three repeated passive records, the lower-platform input acceleration RMS was $\left(1.30\pm 0.11\right)\times 10^{-5}$ g, whereas the upper-platform output acceleration RMS was $\left(3.63\pm 0.58\right)\times 10^{-5}$ g. The corresponding integrated suppression ratio was −8.87±2.12 dB. These results confirm that the passive structure did not provide net isolation over the tested 0.1–10 Hz band; instead, resonance amplification dominated the band-integrated response and established the baseline for the subsequent active-control comparison.

### 4.3. Active-Control Frequency-Domain Suppression

**Figure 11:**
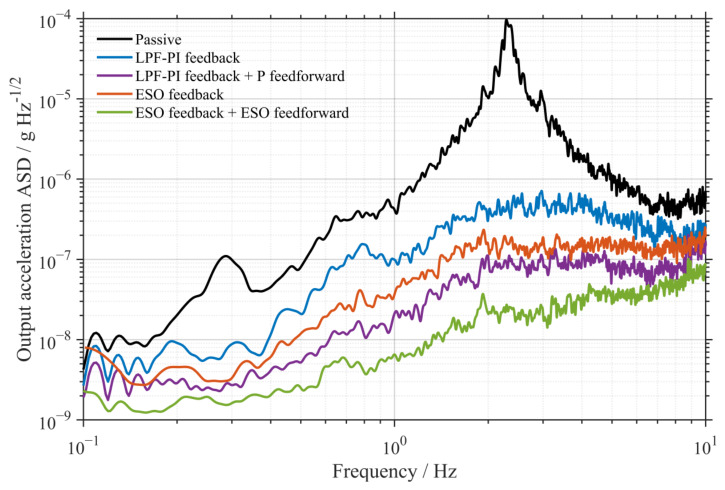
Residual upper-platform acceleration ASD under the passive baseline and four active-control conditions.

**Figure 12:**
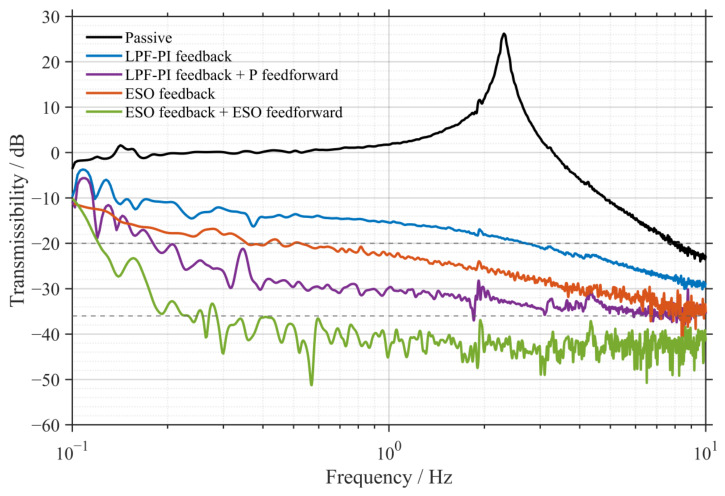
Measured vertical input–output transmissibility of the passive baseline and four active-control conditions.

Accordingly, the performance comparison reflects each complete controller implementation rather than an identical prefilter applied to all active cases. Figure 11 and Figure 12 compare the residual upper-platform acceleration ASD and the input-to-output transmissibility under different active-control conditions. The passive case showed a pronounced peak at the 2.30 Hz vertical resonance. LPF-PI feedback reduced this peak, and proportional lower-platform velocity feedforward further improved the low-frequency attenuation, but both remained higher than the ESO-based cases over much of the 0.1–10 Hz band. ESO feedback provided stronger suppression through the reconstructed velocity and equivalent-displacement feedback states, while the proposed ESO feedback–feedforward condition achieved the lowest output ASD and the strongest transmissibility reduction.

Table 1 summarizes the corresponding quantitative results. The integrated suppression ratio is listed first because it directly quantifies the absolute input–output isolation performance over the 0.1–10 Hz band. Output acceleration RMS and RMS reduction quantify the residual upper-platform vibration level and the improvement relative to the passive baseline.

LPF-PI feedback reduced the output acceleration RMS by 31.53±2.30 dB relative to the passive condition and achieved an integrated suppression ratio of 23.85±0.06 dB. Adding proportional lower-platform velocity feedforward increased this ratio to 33.74±0.21 dB. ESO feedback alone achieved a larger output-RMS reduction of 39.47±1.96 dB and an integrated suppression ratio of 29.83±0.20 dB, showing the benefit of ESO-reconstructed velocity and equivalent-displacement feedback states.

The proposed ESO feedback–feedforward condition gave the best performance, reducing the output acceleration RMS to $\left(1.31\pm 0.09\right)\times 10^{-7}$ g, corresponding to a 48.92±2.12 dB reduction relative to the passive condition. The integrated suppression ratio reached 38.67±0.46 dB, which is 4.93 dB higher than that of LPF-PI feedback plus proportional feedforward. The output RMS was also reduced from $\left(2.78\pm 0.24\right)\times 10^{-7}$ g to $\left(1.31\pm 0.09\right)\times 10^{-7}$ g, and the small standard deviations indicate repeatable improvement under the tested ground-vibration input.

To further avoid relying on improvement relative to the resonant passive baseline, Table 2 reports direct transmissibility magnitudes, defined above, at the 2.30 Hz resonance and over the below-resonance low-frequency band.

The passive structure had a positive transmissibility of 24.89±0.34 dB at the 2.30 Hz resonance, confirming the resonant amplification indicated by the negative integrated suppression ratio. The proposed ESO feedback–feedforward condition reduced the resonance transmissibility to −42.76±1.17 dB. In the 0.1–1 Hz below-resonance band, it also gave the lowest mean transmissibility, −37.79±2.70 dB, compared with −27.71±1.11 dB for LPF-PI feedback plus proportional feedforward. These direct transmissibility metrics confirm that the improvement is not only a reduction relative to the passive output RMS, but also an absolute reduction in upper-platform response with respect to the measured lower-platform input.

**Figure 13:**
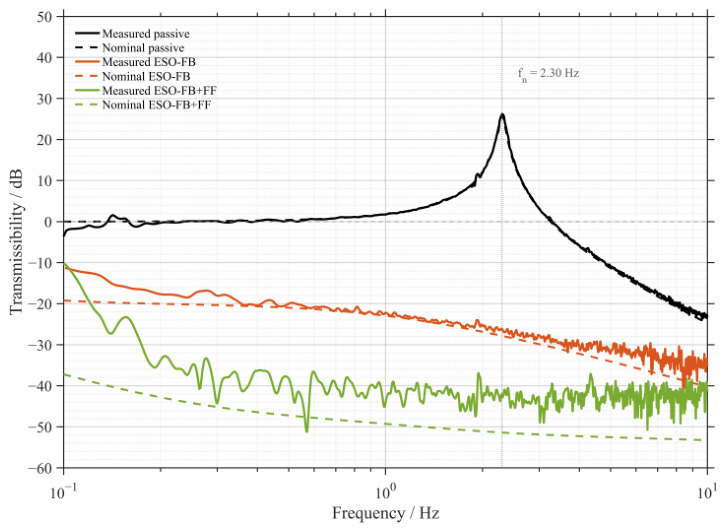
Direct comparison between nominal-model predictions and measured vertical transmissibility for the passive, ESO feedback, and ESO feedback–feedforward cases.

To further evaluate whether the reduced model captures the measured dynamics, Figure 13 compares the nominal-model predictions with the measured transmissibility for three representative cases: passive isolation, ESO feedback, and ESO feedback–feedforward.

The passive and ESO-feedback curves agreed well with the nominal model. Using the same segmented-record calculation as the experimental metrics, their measured-minus-nominal errors at the 2.30 Hz resonance were −0.86±0.48 dB and 1.35±0.37 dB, respectively. The corresponding mean absolute errors were 0.26±0.04 dB and 1.31±0.05 dB in the 0.1–1 Hz band, and 0.59±0.04 dB and 2.76±0.07 dB over the 0.1–10 Hz band. This agreement indicates that the reduced vertical model captures the dominant passive resonance and the feedback-controlled response. The ESO feedback–feedforward case retained lower measured transmissibility than ESO feedback but deviated more from the nominal prediction, with an error of 8.80±1.10 dB at the 2.30 Hz resonance and mean absolute errors of 9.25±2.32 dB in 0.1–1 Hz and 9.75±0.48 dB in 0.1–10 Hz. This larger mismatch is attributed to the greater sensitivity of feedforward cancellation to plant-path matching, actuator gain, sensor-chain phase lag, and residual unmodeled dynamics.

### 4.4. Time-Domain Response of the Best Condition

**Figure 14:**
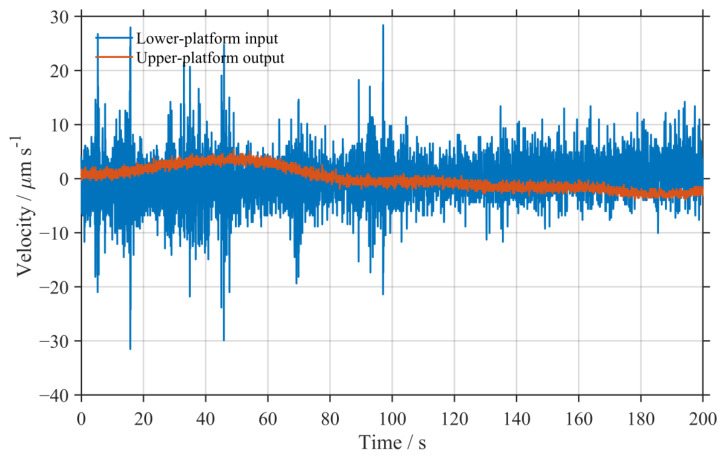
Representative 200 s velocity responses of the lower and upper platforms under the proposed ESO feedback–feedforward condition.

Figure 14 shows a representative 200 s steady-state velocity record under the proposed ESO feedback–feedforward condition. In this record, the measured lower-platform and upper-platform velocity RMS values are $4.598\times 10^{-6}$ m/s and $2.090\times 10^{-6}$ m/s, respectively, corresponding to a record-level velocity RMS reduction of 6.85 dB. For reference, the corresponding peak-to-peak velocity amplitudes are $6.9183\times 10^{-5}$ m/s and $1.0115\times 10^{-5}$ m/s, respectively. These time-domain values provide an amplitude-level illustration of the steady-state response, whereas the band-limited acceleration RMS and integrated input–output suppression ratio in Table 1 are used as the primary quantitative performance measures.

## 5. Conclusions

This study presented a velocity-sensor-based dual-ESO state-reconstruction method for feedforward–feedback low-frequency active micro-vibration isolation. The upper-platform ESO reconstructs the feedback velocity and a bounded equivalent-displacement feedback state, while the lower-platform ESO reconstructs a smoothed lower-platform velocity state for feedforward compensation. These states are incorporated into a composite control law with velocity feedback, equivalent-displacement feedback, and lower-platform feedforward.

Signal-reconstruction simulations verified the intended roles of the two ESOs. The upper-platform ESO preserved the dominant feedback velocity while reducing out-of-band sensor noise by 5.2 dB in the 60–200 Hz band. The lower-platform ESO provided a smoothed feedforward reference with a smoothing index of 21.9 dB, and the equivalent-displacement reconstruction reduced direct-integration drift by 36.3 dB.

Experiments in the vertical 0.1–10 Hz band confirmed the effectiveness of the proposed control structure. Over three repeated 200 s records, the proposed ESO feedback–feedforward condition achieved an integrated input–output suppression ratio of 38.67±0.46 dB and reduced the upper-platform output acceleration RMS to $\left(1.31\pm 0.09\right)\times 10^{-7}$ g, corresponding to a 48.92±2.12 dB reduction relative to the passive condition. The direct transmissibility and nominal–measured comparisons further confirmed that the improvement was evaluated with respect to the lower-platform input and that the reduced model captured the dominant passive and feedback-controlled dynamics. These results demonstrate that low-frequency active micro-vibration isolation can be achieved using only velocity measurements, without an additional displacement sensor.

The present validation is limited to the vertical direction and fixed controller parameters for the tested payload and excitation level. Future work will extend the method to multi-axis isolation, long-term operation under varying excitation conditions, and adaptive parameter tuning.

## Figures and Tables

**Table 1:** Experimental isolation performance under different control strategies. Values are mean ± standard deviation over three repeated 200 s records.

Condition	$\eta_{0.1-10}$/dB	Output RMS/g	RMS Reduction/dB
Passive	−8.87±2.12	$\left(3.63\pm 0.58\right)\times 10^{-5}$	0.00±0.00
LPF-PI feedback	23.85±0.06	$\left(9.77\pm 1.59\right)\times 10^{-7}$	31.53±2.30
LPF-PI feedback + P feedforward	33.74±0.21	$\left(2.78\pm 0.24\right)\times 10^{-7}$	42.40±2.22
ESO feedback	29.83±0.20	$\left(3.88\pm 0.18\right)\times 10^{-7}$	39.47±1.96
ESO feedback + ESO feedforward	38.67±0.46	$\left(1.31\pm 0.09\right)\times 10^{-7}$	48.92±2.12

**Table 2:** Direct input–output dB-transmissibility comparison at resonance and in the low-frequency bands. Values are mean ± standard deviation.

Condition	$T_{\mathrm{dB}}$ at 2.30 Hz/dB	Mean $T_{\mathrm{dB}}$, 0.1–1 Hz/dB	Mean $T_{\mathrm{dB}}$, 0.1–10 Hz/dB
Passive	24.89±0.34	0.42±0.02	−8.30±0.04
LPF-PI feedback	−19.00±0.10	−13.24±0.25	−23.14±0.08
LPF-PI feedback + P feedforward	−32.82±0.34	−27.71±1.11	−33.59±0.05
ESO feedback	−26.45±0.31	−19.92±0.06	−30.00±0.08
ESO feedback + ESO feedforward	−42.76±1.17	−37.79±2.70	−42.12±0.52

## Data Availability

The data presented in this study are available from the corresponding author upon reasonable request.

## Abbreviations

The following abbreviations are used in this manuscript:

ADRC	Active disturbance rejection control
ASD	Amplitude spectral density
ESO	Extended state observer
FRF	Frequency response function
LPF-PI	Low-pass-filtered proportional–integral
RMS	Root mean square
